# Effects of Peptone Supplementation in Different Culture Media on Growth, Metabolic Pathway and Productivity of CHO DG44 Cells; a New Insight into Amino Acid Profiles

**DOI:** 10.7508/ibj.2015.04.002

**Published:** 2015-10

**Authors:** Fatemeh Davami, Farnaz Eghbalpour, Leila Nematollahi, Farzaneh Barkhordari, Fereidoun Mahboudi

**Affiliations:** 1 *Biotechnology Research Center, Pasteur Institute of Iran, Tehran, Iran; *; 2 *Science and Research Branch, Islamic Azad University of Arak, Markazi Province, Iran*

**Keywords:** CHO cells, Culture media, Peptones, Recombinant proteins

## Abstract

**Background:**

The optimization of bioprocess conditions towards improved growth profile and productivity yield is considered of great importance in biopharmaceutical manufacturing. Peptones as efficient sources of nutrients have been studied for their effect on media development; however, their role on metabolic pathway is not well understood.

**Methods:**

In the present study, the effect of different concentration of peptones on a recombinant Chinese hamster ovary (CHO) cell line grown in three serum-free suspension cultures was determined. Six peptones of different origins and available amino acid profiles were investigated regarding their impact on cell growth, productivity, and metabolic pathways changes.

**Results:**

In optimized feeding strategies, increases of 136% and 159% in volumetric productivity (for a low-nutrient culture media) and 55% (for a high-nutrient culture media) were achieved. Furthermore, particular sources of peptones with specific amino acid profile developed preferential results for each different culture medium. Two peptones, SoyA2SC and SoyE-110, were the only hydrolysates that showed production improvement in all three media. Casein Peptone plus Tryptone N1 and SoyA3SC showed different improved results based on their implemented concentration for each individual basal medium.

**Conclusion:**

The amino acid profile of peptones may provide clues to identify the most effective feeding strategies for recombinant CHO cells.

## INTRODUCTION

Mammalian expression systems are the most prevalently used hosts in recombinant protein production, due to their appropriate post-translational modifications. Nowadays, almost 60-70% of recombinant proteins are successfully produced in Chinese hamster ovary (CHO) cells mainly due to their correct post-translational modifications, and relatively easy adaptation to suspension growth in serum-free media for industrial scale bioreactors. The optimization of cell culture conditions towards improved growth and productivity of recombinant CHO (rCHO) cells is a critical step in biopharma-ceutical process development and still faces a series of problems. 

Commercial production of therapeutic proteins is now mainly based on serum-free media due to its economical advantages, beneficial effect on the downstream processing, and biological safety from infectious contaminants. Nevertheless, development of an optimized culture and medium condition has been a challenging debate and still no universal serum-free media applicable to all cell lines is available [1, 2]. 

 Recently, quite a large number of researches have been devoted to predictable media optimization with respect to its commercial outcomes for the biopharmaceutical industry.

 Supplementation of hydrolysates has been shown to enhance cell growth and/or productivity in mammalian cells, such as rCHO cells [3, 4], hybridoma [5], HEK293 cells [6] BHK cells [1], and insect cells [7].

 Peptones are water-soluble protein hydrolysates, containing peptides, amino acids, and inorganic salts as well as other compounds, such as lipids, vitamins, and sugars [5]. Plant-derived peptones can improve cell growth and enhance specific and volumetric productivity both in stable and transient expression systems [6, 8, 9] . 

The mechanism of peptones’ positive effects is not well understood. Previous studies have shown that they are substantial sources of nutrients and can provide cells with anti-apoptotic functions [10] as well as an stimulator in transfection efficiency of HEK293 cells [8].

 However, the impact of peptone supplementation on metabolic behavior of rCHO cells is not properly known [11-13]. Furthermore, it is also unclear how each specific amino acid profile of a peptone is capable of altering metabolic attitude of the cells towards more glucose consumption or lactate and ammonia production. Therefore, the positive effect of peptones is still unpredictable and similar feeding strategies may cause different responses in various systems [14-16]. Consequently, a peptone-supplemented bioprocess needs to be optimized for a specific cell line or clone.

The ultimate aim at biopharma industry is to obtain optimized growth profile of cells along with higher yields of therapeutic protein production. Therefore, further understanding of intercellular changes during media supplementation is a promising approach for a predictable knowledge-based media development, which can lead to constancy in biopharma [17-20].

**Fig. 1:**
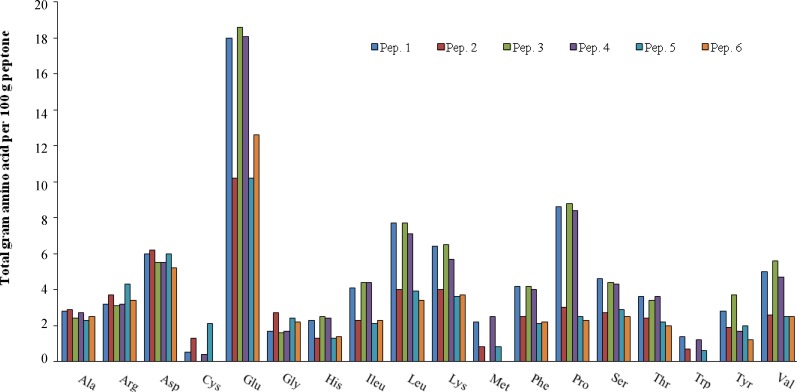
Amino acid profile differences in casein and soy peptones (Pep) based on total amino acid content (g) per 100 g of peptone

In the present study, a few plant-derived and casein-derived peptones (Fig. 1 and Table 1) were investigated for their amino acid profile correlation with growth, productivity, biomass, and alterations in cell metabolic attitudes of a rCHO cell line. 

## MATERIALS AND METHODS

***Media and reagents.*** The two proprietary serum-free media used were denominated CD DG44 and ProCHO 5 from Invitrogen (GIBCO Invitrogen, USA) and Lonza (Verviers, Belgium), respectively. The media were supplemented with 13.6 mg hypoxanthine l-1, 3.9 mg thymidine l-1, and 4 mM and 6 mM glutamine for ProCHO 5 and CD DG44 respectively. Furthermore, a basal medium based on RPMI 1640 (BRC-CD medium [BRC CDM]) was developed in the laboratory and supplemented with 44 mM glucose and 6 mM glutamine. All medium supplements used in this study were purchased from Sigma-Aldrich (St. Louis, MO, USA). Chromolize t-PA (tissue plasminogen activator**) **Assay Kit was purchased from Biopool (Trinity Biotech PLC, Ireland). Packed cell volume (PCV) tubes and tube-spines were from (TPP, Techno Plastic Products AG, Trasadingen, Switzerland). Peptones were supplied from Organotechnie (La Courneuve, France) providing the total amino acid composition, molecular weight distribution, and free amino acid content of the peptones (Fig. 1 and Table 1). Peptone stock solutions were prepared (20%, w/v), sterilized by filtration through 0.2 µm media filters, and stored at 4ºC.

***Cell cultivation.*** The stable CHO DG44-derived cell lines, t-PA-producing cells from our previous studies [21-23]), were used in this study. Cultures were agitated at 110 rpm in TubeSpin® Bioreactors on an orbital shaker (at 37ºC in a % CO_2_ atmosphere [24]. The cultures were inoculated with cells from the mid- exponential growth phase at a cell concentration of 0.20 × 10^6^ cells/ml. On the day of peptone addition, cells were centrifuged and transferred to 5 ml fresh medium (CD DG44/ProCHO 5/BRC-CDM) containing a specific amount of a peptone in TubeSpin® Bioreactor 50 tubes (TPP). The cultures were agitated at 110 rpm on an ISF-4-W orbital shaker at 37ºC in humidified 5% CO_2_ atmosphere.

**Table 1:** Total amino acid content, average molecular weight (MW), and MW distribution of the peptones evaluated in this study

**Peptone** a	**Catalogue** **No.**	**Origin**	**Name**	**Total amino acid content (g/100 g)**	**Average MW (daltons)**		**MW distribution (%)**			
							**<0.3** **kDa**	**0.3-1** **kDa**	**1-10 kDa**	**>10 kDa**
1	19544	casein	Casein Peptone Plus	85.1	491		38.5	53.0	8.5	0.0
2	19685	soy	Soy Peptone A3 SC	55.2	227		56.0	41.4	2.6	0.0
3	19546	casein	Casein Peptone E1	82.4	840		23.5	48.6	27.8	0.1
4	19553	casein	Tryptone N1	81.6	490		31.7	60.1	8.2	0.0
5	19649	soy	Soy Peptone A2 SC	53.8	503		30.6	60.8	8.6	0.0
6	19885	soy	Soy Peptone E-110	49.4	1206		31.1	48.7	18.5	1.9

a Data available from Organotechnie (www.organotechnie.com)

***Cell number. ***Cell density and viability were assessed by the Trypan blue dye exclusion method using a hemocytometer (Neubauer improved, Brand). Cell viability was determined by the Trypan blue exclusion method (1:1 mixture of 0.2% trypan blue in a normal saline and cell sample). After cell counting, the remainder of each sample was centrifuged (at 5000 ×g for 1 min) to remove the cells, and the supernatant was frozen for further protein production and metabolite consumption/production rate analysis.

***Biomass determination. ***Biomass was determined by the PCV method using PCV tubes (TPP, Techno Plastic Products AG, Trasadingen, Switzerland) [25]. A cell density of 1 × 10^6^ cells ml-1 was equivalent to a PCV of 0.25% for cells under standard cultivation conditions at 37°C.

***Metabolite determinations. ***Glucose measurement was performed based on an enzymatic colorimetric method with a glucose oxidase kit (Pars Azmun Inc., Iran). Lactate determination was based on Bergmeyer's technique [26] at wavelength of 340 nm via a lactate dehydrogenase-based spectrophotometric kit (Pars Azmun Inc., Iran) [27]. Ammonium concentrations were determined by specialized urea (ammonia) assay kits (Pars Azmun Inc., Iran). 

***Quantification (amidolytic activity test). ***Biopool's Chromolize t-PA Assay Kit is a biofunctional immunosorbent assay based on capturing t-PA by sp-322 monoclonal antibodies coated on the microtest wells. After fulfilling, all the steps from the kit’s manual samples were read at 405 nm and 492 nm. Absorbance at 492 nm was measured and subtracted from 405 nm. Various dilutions of each sample were assayed. The amount of developed color is proportional to the amount of t-PA activity in the sample. Results are presented in amidolytic international that are defined as the amount of plasminogen activator required to release 1 pmol of p-nitroanilide in 1 minute at 24°C at 405 nm in a I-cm path length, using an extinction coefficient of 9629 mol/L-' cm-' for p-nitroanilide.

## RESULTS

**Fig. 2:**
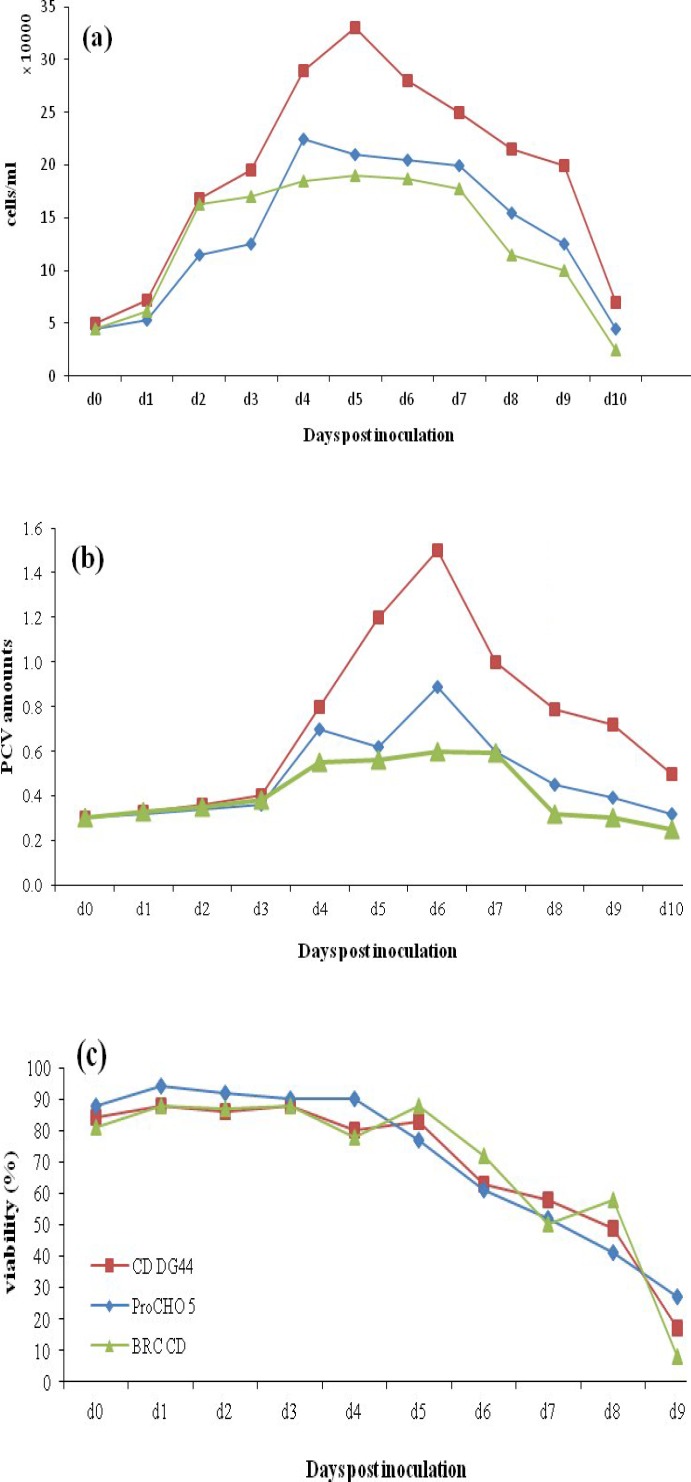
Viable cell numbers (a), PCV amounts (b), and viability (c) for clone 2 cultivated in ProCHO 5, CD DG44, and BRC-CDM

*Determination of CHO DG44 cell growth profile in different media.* To investigate media dependency of growth profile, the CHO clone was simultaneously cultivated in the same cultivation condition in three different chemically defined media, CD DG44, ProCHO 5, and our home-made BRC-CDM. Figure 2 show the growth profile of t-PA producing CHO DG44 cells based on cell count, viability, and PCV amounts. As shown in Figure 2, higher cell densities (Fig. 2a) and higher amounts of PCV (Fig. 2b) were achieved in ProCHO 5 medium. Furthermore, cells tend to show almost similar growth in CD DG44 and BRC-CDM in terms of cell densities and biomass with slightly better results for CD DG44. Maximum cell density was on day 7 and culture duration was 11 days but after day 6, the viability of the cells was started to reduce (Fig. 2c). The same comparisons was made for CHO DG44 non-transfected cells in three media, and the growth profile was almost the same, except for slightly fewer maximum cell densities for non-transfected cells and the downward shift in viability, which for t-PA producing CHO DG44 takes place at day 7 and for non-transfected cells at day 9 (data not shown).

**Fig. 3:**
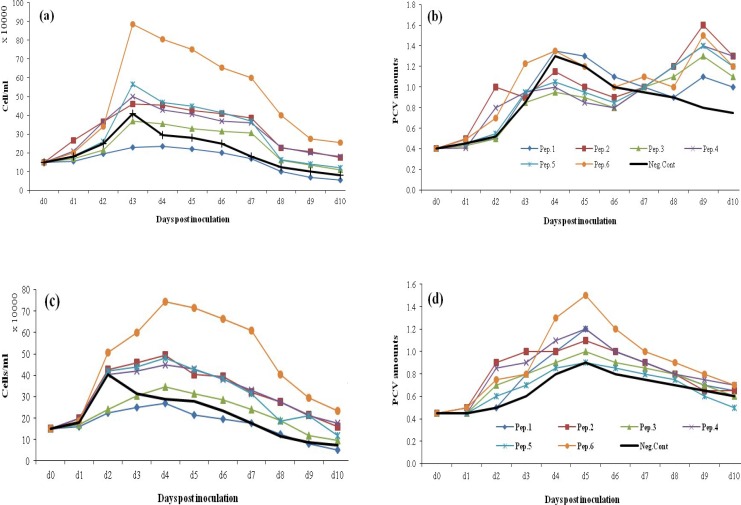
The effect of 1 and 2 gl^-1^ peptone (Pep) concentrations on cell density and PCV values of t-PA producing CHO DG44 cell line cultivated in ProCHO 5 medium. Viable cell numbers and PCV values in 1 gl^-1^ (a and b) and 2 gl^-^^1^ (c and d) peptone concentration.

**Fig. 4:**
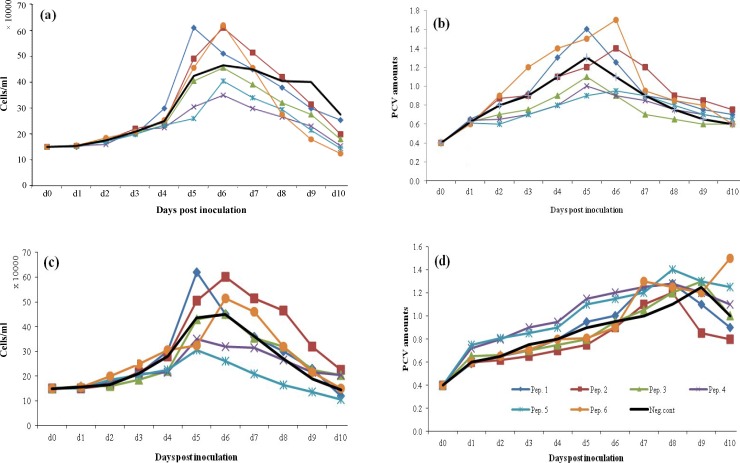
The effect of 1 and 2 gl^-1^ peptone (Pep) concentrations on cell density and PCV values of t-PA producing CHO DG44 cell line cultivated in CD DG44 medium. Viable cell numbers and PCV values in 1 gl^-1 ^(a and b) and 2 gl^-1 ^(c and d) peptone concentration

**Fig. 5:**
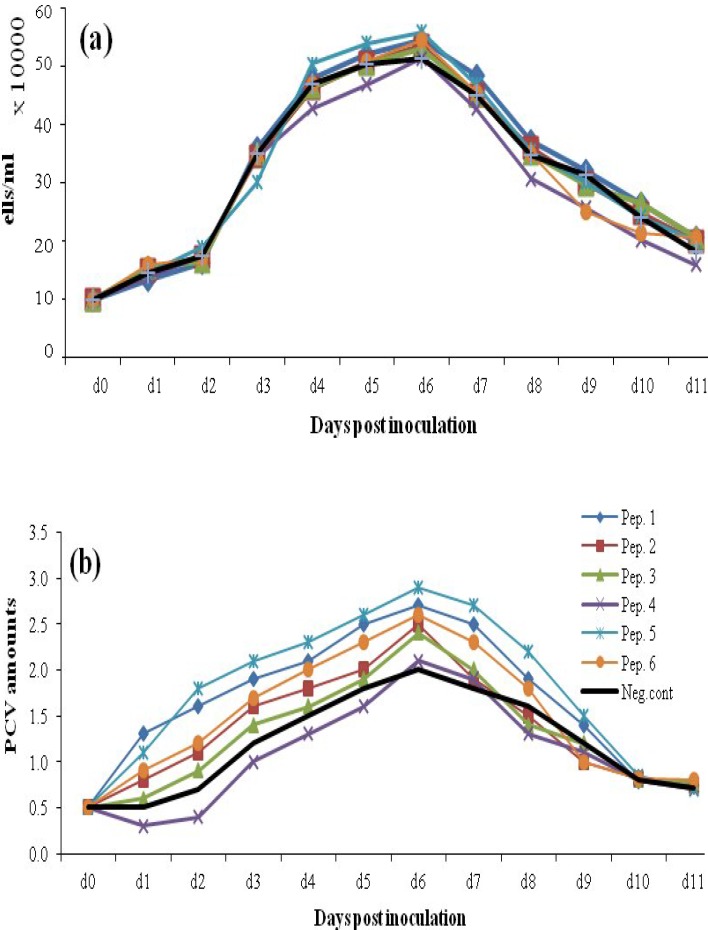
The effect of 1 gl^-1^ peptone (Pep) concentrations on cell density and PCV values of t-PA producing CHO DG44 cell line cultivated in BRC-CDM. **(a) **Viable cell numbers, (b) PCV values

*Media dependency of peptone effect in different concentrations. *T-PA producing CHO DG44 cells were cultivated in three different chemically defined media (ProCHO 5/CD DG44/BRC) to which individual peptones were added with concentrations of 1 and 2 gl^-1^. For ProCHO 5 medium, peptone supplementation resulted in a biomass growth in each of the supplemented peptones (Fig. 3). Regarding cell densities in both feeding concentrations, only peptone one in spite of a rise in PCV amounts led to reduced cell densities compared to control group. In 1 gl^-1 ^feeding concentration, peptones 6, 5, 2, 4, and 3 showed an apparent rise in maximum cell densities up to 115, 59, 54, 45, and 20%, respectively (Fig. 3a and 3b). This growth in cell numbers was even more drastic in 2 gl^-1 ^feeding strategy; 156, 70, 65, 55, and 18% for peptones 6, 2, 5, 4, and 3, respectively (Fig. 3c and 3d). PCV values made significant rises with both peptone feeding strategies except for peptone 5, which led to reduced biomass in 2 gl^-1^. In CD DG44 medium supplemented with both 1 and 2 gl^-1 ^of peptones (Fig. 4a and 4b), the positive impact of peptones on cell densities and biomass improvement was observed with peptones 1, 2, and 6. Maximum rise in cell densities was achieved with peptone 1 (43% increase for both feeding concentrations), followed by peptones 2 and 6. Regarding PCV values, 1 gl^-1^ of peptone 6 resulted in highest improved amounts of 54%, followed by peptones 2 (27%) and 1 (23%). Concerning 2 gl^-1^ feeding, 72, 61, and 23% growth in PCV values was achieved with peptones 2, 6, and 1, respectively (Fig. 4c and 4d). As illustrated in Figure 5a and 5b, in BRC-CDM containing 1 gl^-1 ^peptone concentration, except for peptone 4, other five peptones showed biomass increases. A rise in PCV amount was 45, 35, 30, 25, and 20% for each of the peptones 5, 1, 6, 2, and 3, respectively. However, the viable cell densities and viability did not improve to great extent. Peptone 4 was the only supplement with no significant effect on BRC- CDM. The results for 2 gl^-1 ^were not outstanding (data not shown).

**Fig. 6:**
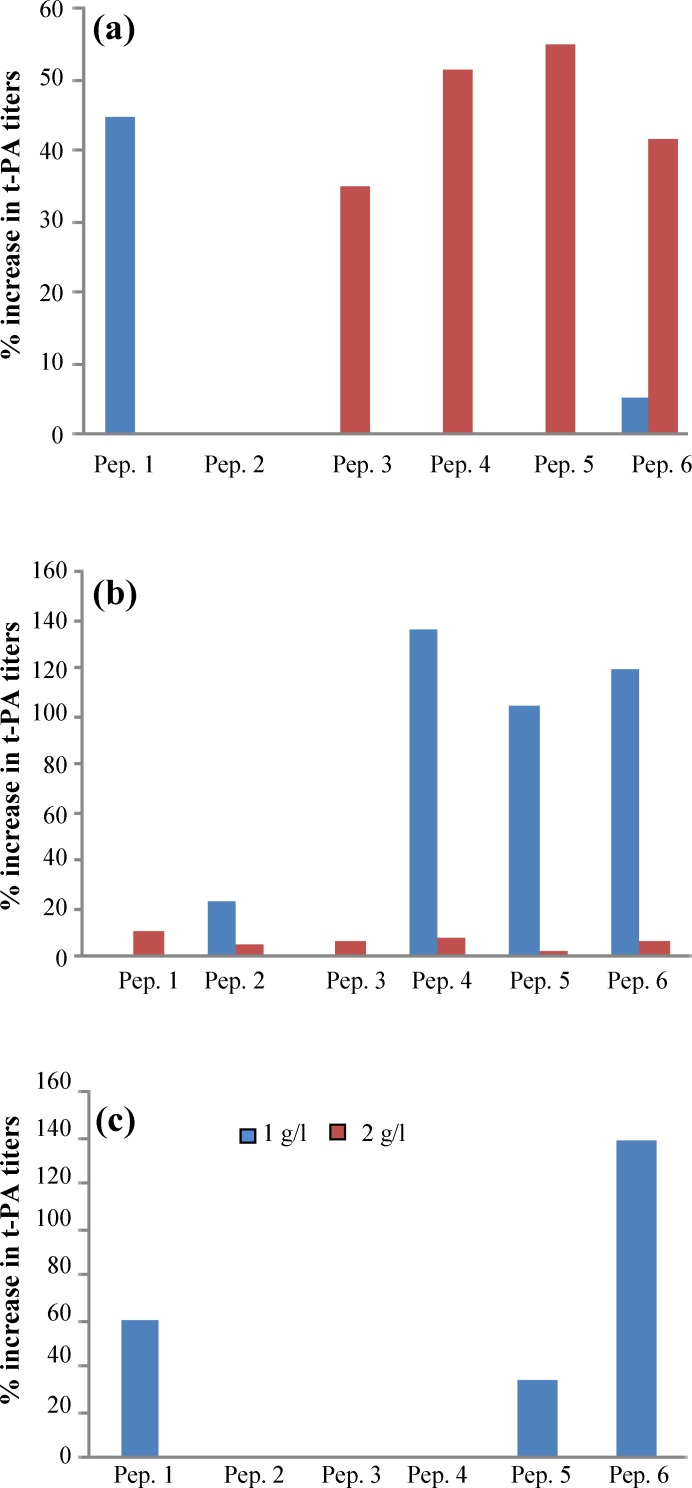
Percentage of increase in t-PA titers on day nine with 1 and 2 gl^-1 ^peptone (Pep) concentrations. ProCHO 5 (a), CD DG44 (b), and BRC-CDM (c) media supplemented with peptones

*Determination of the peptones effects on cell productivity. *As mentioned above, the Chromolize t-PA assay ELISA based kit was used to determine the amount of total active form of t-PA protein. For quantitative determination of human t-PA activity in supernatant samples of transfected CHO cell, a biofunctional immunosorbent assay was performed. Based on Biopool’s Chromolize t-PA assay kit, amidolytic unit was measured on day 9 of each culture. In ProCHO 5 medium with 2 gl^-1 ^peptone concentration, peptones 5, 4, 6, and 3 showed a drastic rise in accumulated proteins on day 9 up to 55%, 51%, 41% and 35%, respectively. In 1 gl^-1^, only peptone 1 represented an increased amount of 44% productivity on day 9, while the results for other peptones were either slight or even negative (Fig. 6a). On the other hand, in CD DG44 medium, 1 gl^-1 ^concentration of peptones 4, 5, 6, and 2 could result in 136, 120, 104, and 22% increase in t-PA titers, while no significant improving results were seen in 2 gl^-1 ^feeding concentration (Fig. 6b). As represented in Fig. 6c, for BRC-CDM, with 1 gl^-1 ^concentration, peptones 6, 1, and 5 were capable to show increase of 139%, 60.36% and 33.64% for day 9 production yield respectively and the same as CD DG44, 2 gl^-1 ^feeding concentration did not affect productivity. 

***Determination of metabolic behavior of t-PA producing CHO DG44 cells:***

**Fig. 7:**
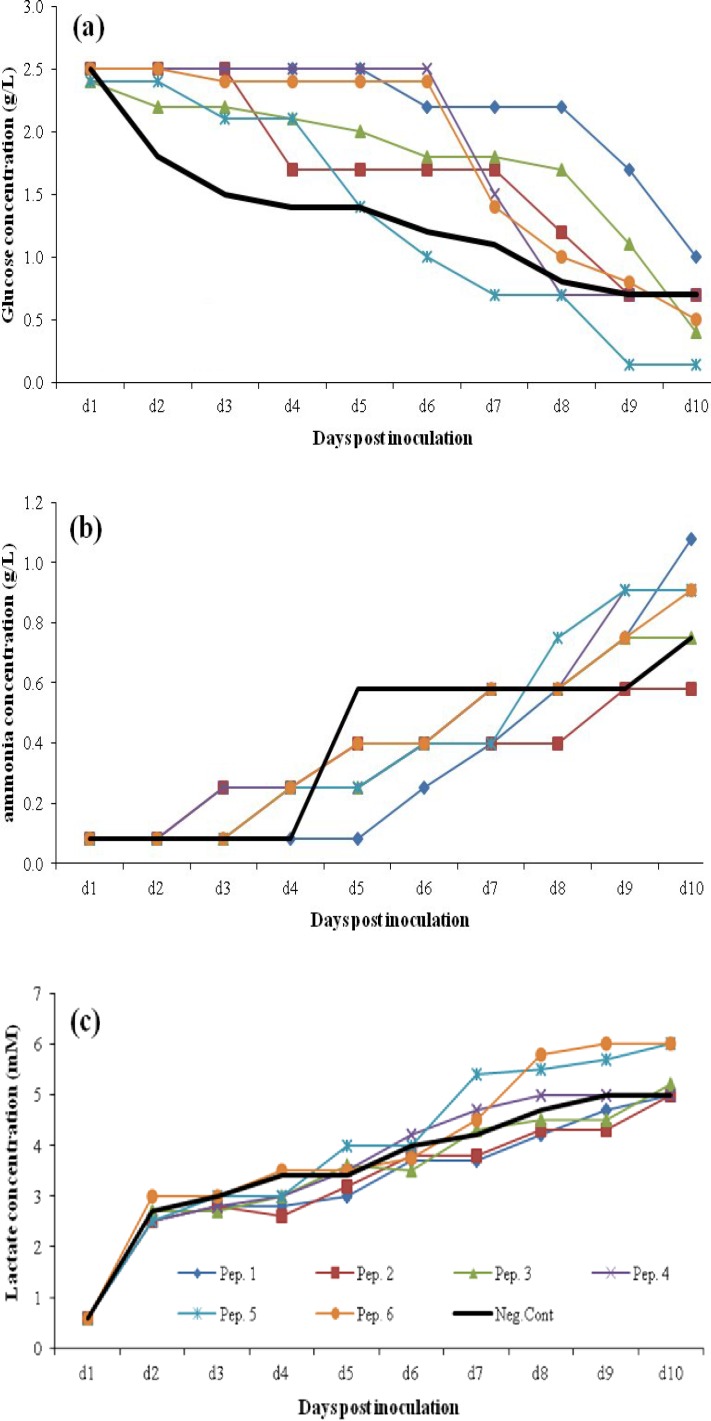
Metabolic behavior changes in fed-batch cultures of the CHO cells using 2 gl^-1^ peptone (Pep) supplementations in ProCHO 5 medium. (a) Glucose concentrations, (b) ammonium concentrations, (c) lactate concentrations

***Metabolic changes in ProCHO 5 medium.*** Figure 7 illustrates the methabolic profile of rCHO cell lines in the presence of 2 gl^-1^ supplementation of peptones for ProCHO 5 medium. When utilized in 2 gl^-1^ concentrations, all six peptones maintained cells in higher amounts of glucose during culture durartion (Fig. 7a). In contrast, with 1 gl^-1 ^concentration, peptones 1, 2, and 6 were able to supply cells with higher amounts of glucose during culture (data not shown). Regarding ammonia production (Fig. 7b), more fluctuating results were observed with different peptone supplements compared to control negative samples. The average ammonia accumulation on day 10 of culture was 0.51 and 0.85 gl^-1^ with 1 and 2 gl^-1^ peptone feeding concentrations, respectively. Lactate byproduct accumulation at the end of culture was greater with peptones 4, 5, and 6 (Fig. 7c). Interestingly, a similar pattern of lactate production and final cumulative amounts were observed in two peptone supplement concentrations of 1 and 2 gl^-1^; 5.03 and 5.3 mM average final lactate concentrations, respectively. 

**Fig. 8:**
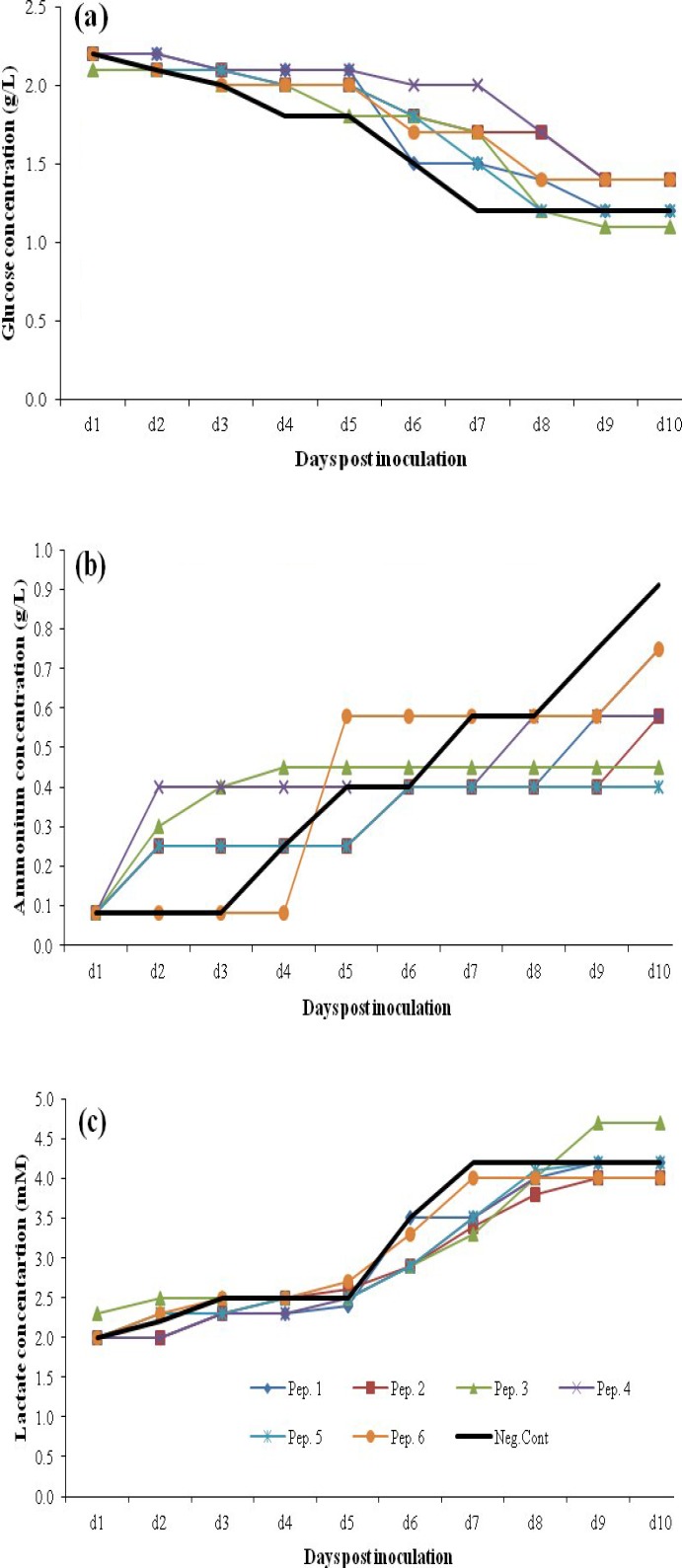
Metabolic behavior changes in fed-batch cultures of the CHO cells using 1 gl^-1^ peptone(Pep) supplementations in CD DG44 medium. (a) Glucose concentrations, (b) ammonium concentrations, (c) lactate concentrations

***Metabolic changes in CD DG44 medium.*** Figure 8 represents the metabolic profile changes in CD DG44 medium supplemented with 1 gl^-1 ^of peptone concentration which was the selected feeding amount in terms of production titers in this media. Higher maintained glucose concentrations (Fig. 8a) were observed with all six peptones in both 1 and 2 gl^-1 ^(data for 2 gl^-1 ^not presented). In terms of ammonia production (Fig. 8b), peptone supplements resulted in higher ammonia concentartions up to day 6 with a shift to less ammonia amounts from day 6 to 10. The average ammonia accomulation on day 10 was 0.58 and 0.83 gl^-1^ with 1 and 2 gl^-1^ peptone feeding concentrations, respectively. Figure 8c shows the lactate production pattern during the culture. Except for peptone 3, all other five peptone supplements produced less lacatate amounts in 1 gl^-1^. Final average lactate accomulation on day 10 were 4.18 and 5.46 mM in 1 and 2 gl^-1^ feeding concentrations. 

**Fig. 9:**
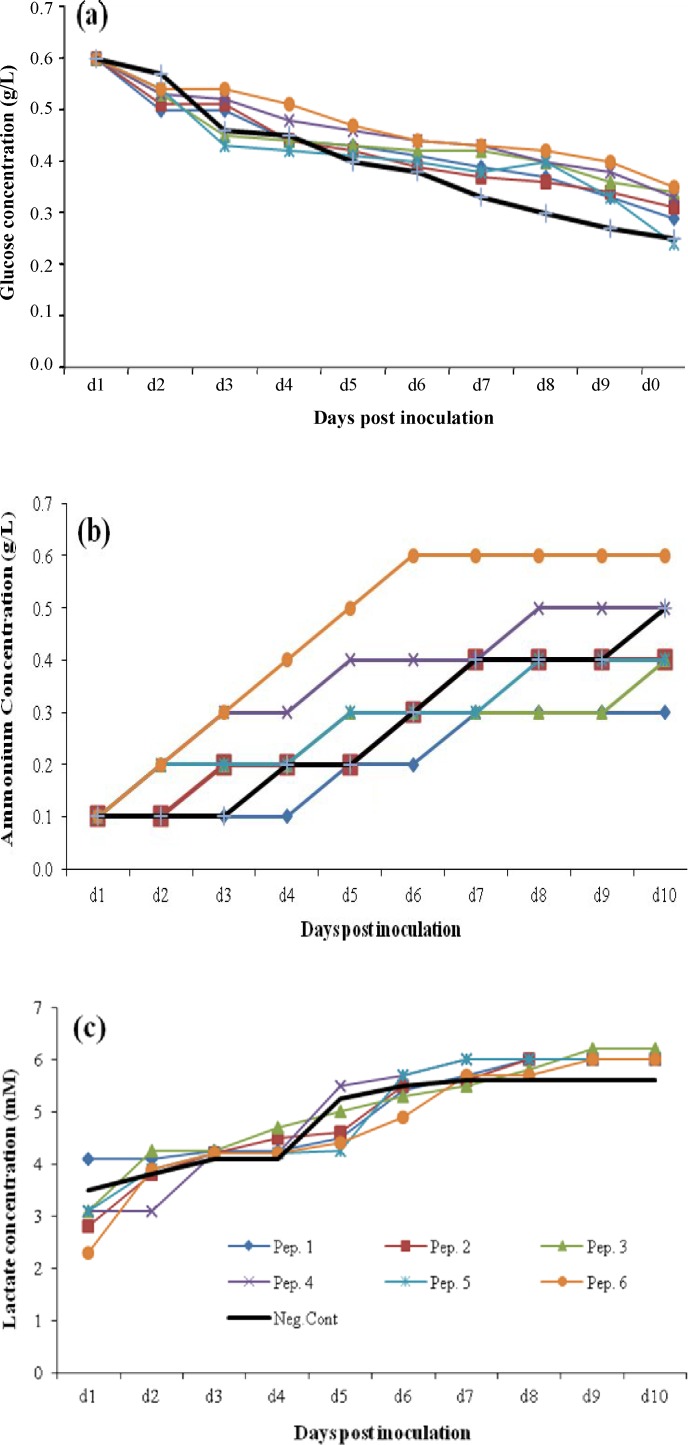
Metabolic behavior changes in fed-batch cultures of the CHO cells using 1 gl^-1^ peptone (Pep) supplementations in CD DG44 medium. (a) Glucose concentrations, (b) ammonium concentrations, (C) lactate concentrations

***Metabolic changes in BRC-CDM. ***The metabolic profiles of rCHO cells in BRC-CDM supplemented with 1 gl^-1^ of different peptones are represented in Figure 9. This concentration was the selected feeding strategy, which led to better production amounts. No great effect on glucose consumption was observed with peptones feeding in this medium (Fig. 9a). However, in case of ammonia production (Fig. 9b), in spite of higher cumulative amounts with peptones 4 and 6, the average ammonia concentration on day 10 of culture with peptone feeding was reduced to 0.43 gl^-1^ compared to control non-fed culture (0.5 gl^-1)^ .

 No obvious change in lactate accomulation pattern with feeding peptones was observed in BRC-CDM (Fig. 9c). However, the final average amounts was slightly higher with peptone feeding compared to control samples (6.03 mM compared to 5.6 mM).

## DISCUSSION

Peptones are widely used as supplements for serum-free culture media. The growth-promoting activity of peptones may have a dual effect in batch cultures, while it may promote rapid cell growth in the first days of culture. Also, it may lead to early depletion of vital nutrients and concomitant release of toxic metabolites, more rapid decline of culture viability, apoptosis and release of proteases which may degrade the product. In industrial processes, various strategies aiming to decrease cell growth during the production phase are routinely applied [28].

Similar studies on hydrolysate supplementation strategy have mainly focused on using statistical methods such as design of experiment (DoE) to determine the best mixture ratio to enhance productivity [29] regardless of peptones different amino acid profile. Thus, they have not considered the underlying story and need to be optimized for each protein and producing cell individually. However, the aim of the current study and the few similar papers [30] is to find a relationship between amino acid profile and production profile changes during media supplementation.

In a recent report, a proteomic approach was applied to identify CHO cells intracellular protein with either induced or suppressed expression upon peptones feeding [31]. The expression of several proteins involved in cell proliferation, metabolism, and protein folding/secretion was induced by peptone supplementation. However, other proteins involved in growth arrest and apoptosis induction were down- regulated, suggesting that the growth-promoting effect of peptones acts through multiple molecular targets. These data supports that peptone impact on metabolic shift can also be traced in metabolic shifts of recombinant cells.

Although the molecular mechanisms of growth promoting effects of peptones are not fully understood, the positive effects observed could be considered as a consequence of the diverse amino acid composition of the peptones [32-35]. However, the relation between their amino acid content and their role in growth, production, and metabolic behavior of recombinant cells are poorly understood. 

Here, we propose that peptone supplementation effects are correlated to amino acid profile, which resulted in metabolic behavior alterations during cultures supplemented with peptones. The peptones used in this study were intentionally chosen among commercially available peptones of plant and casein origin with available total and free amino acid content and molecular distribution on the ground that a correlation exists between amino acid profile and the resulted cell attitude. This approach would be a good start point for further detailed analysis, determination and as a final goal prediction of the best strategy for media optimization. The effect of peptone-mediated improvement on a basal serum-free medium was analyzed by assessing the growth, productivity, and metabolic behavior of a rCHO clone.

The growth and productivity promoting effect of peptones is dependent on the basal medium nutrient composition. The results of cultivation in three different basal media (ProCHO 5, CD DG44, and BRC CD) showed that t-PA producing CHO cell line represents privilege for ProCHO 5 compared to CD DG44 and BRC-CDM in terms of maximum achieved cell densities and biomass amount (Fig. 2). This data is convincing enough because ProCHO 5 as a high-nutrient medium is optimized for recombinant protein production in suspension adapted cells. On the other hand, CD DG44 and BRC CD (our house-made) as low-nutrient media show a similar growth profile, which is significantly lower in terms of cell densities and biomass amounts. Peptone supplementation strategies are supposed to show more distinguished effects in a low-nutrient medium compared to a high-nutrient one.

Determining the optimum peptone feeding concentration is a complicated step towards media supplementation. Our data suggests that a specific peptone concentration should be set for each basal medium. As shown in Figures 3, 4, 5, and 6, for a high-nutrient medium such as ProCHO 5, the higher concentration of peptones (2 gl^-1^) tend to have higher production-promoting effects. Also regarding cell densities, 2 gl^-1 ^is the concentration of choice in this medium with higher cell densities achieved in comparison to 1 gl^-1^. In contrast, for low-nutrient media, such as CD DG44 and BRC CD, 1 gl^-1 ^concentration leads to sharper effects. Concerning  protein production titers, both media showed distinctly improved results only in 1 gl^-1^. 

These results also confirm that while focusing on production titers, a definite set of peptones shows a privilege in each basal medium, which is totally different in terms of prioritization. In other words, in ProCHO 5, peptones 5, 4, 6, and 3 are selected and prioritized in the mentioned order, while in CD DG4, the order goes for 4, 6, 5, and 2 and in BRC CD is 5, 1, 6, and 2. The story behind these differences is the key factor for predicted media optimization. 

In the current study, we observed that peptones from the same source casein/soy do not necessarily produce similar results for a specific media. In ProCHO 5 medium for example, two casein-based peptones (peptones 3 and 4) and two soy-based peptones (peptones 5 and 6) showed an apparent increase in productivity. In CD DG44, all the three soy peptones (6, 5, and 2) were preferred but one of the cases in peptones (4) was able to result in higher production titers. BRC-CDM is preferably enriched with soy peptones (6 and 5) and one of the cases in origins (peptone1). This led us to have a closer look at amino acid profile of each of the six peptones.

 Peptone 1 which shows effect in BRC CD is different from other casein peptones in its aspartic acid content (Fig. 1), which is more close to soy peptone origins rather than casein ones. The selected casein peptone for CD DG44, peptone 4, is different from other casein peptones regarding its higher methionine amounts. 

It is worth mentioning that in ProCHO 5 medium when peptones are utilized in a low concentration (1 gl^-1^), only peptone 1 with high aspartic acid can result in improving production yields. This peptone shows its effect with biomass increase in spite of reduced cell numbers. It also provides the cells with improved metabolic profile, reduced glucose consumption, and ammonia and lactate production (data not shown). On the other hand, peptone 6 in this concentration (1 gl^-1^) reaches much higher cell densities with a sharp reduction in glucose consumption. Nevertheless, it is not able to give a drastic rise to production titers, which may be due to high amounts of lactate produced (data not shown). Furthermore, it was observed that 2 gl^-1^ peptones as a source of nitrogen, produce average amounts of 0.85 gl^-1^ of ammonia byproduct on day 10, which is above the inhibitory range (0.6 gl^-1^) [36]. However, these supplements are still capable of production improvements in this concentration (Figs. 6 and 7).

 Regarding CD DG44 medium with 1 gl^-1^, all peptones except for peptone 3 reduce glucose consumption rate during culture. Moreover, this is the only peptone that produces a high amount of lactate byproduct in comparison to the other peptones. Peptone 3 is different from other casein-based peptones in which it lacks cysteine, methionine, and tryptophan. This peptone also contains some amounts of glutamine, anserine, and citrulline. These differences are possibly the reason for its various effects on metabolic pathway and its negative impact on protein production in this media. 

In BRC CD besides two soy peptones (5 and 6), the “casein peptone plus” with highly different aspartic acid amount affects cells productivity in a positive manner. This medium as the less nutrient basal medium results in the maximum production increase effect of 159%, while in CD DG44 cells, the greatest titer rise is 136%. Except for higher accumulation of ammonia, no drastic change in metabolic attitude was observed with peptone supplementation in this special medium. It is also interesting to stress that peptone supplementation is not the reason for reduced cell proliferation or productivity since the accumulation of ammonia and lactate is less than the levels reported in the literature as being inhibitory (18 mM of lactate [37] and 8 mM of ammonia [36, 38]). Based on previous studies, positive effect of peptones on the production pattern in t-PA producing CHO cells may be attributed to scavenging of proteolytic activities produced by the cells from the peptone peptides [39]. 

 In this study, it was observed that peptone 6, a soy-based peptone with a higher amount of glutamic acid compared to other soy peptones (Fig. 1), and also composed of some amounts of glutamine, asparagines, and citrulline, is the only peptone improving productivity with all media and in every two concentrations used. This data corroborates previous investigations on improving effect of glutamine in t-PA production [39, 40].

High ammonia concentration (up to 7.5 mM) has been shown to have negative impact on t-PA production [38, 41]. However, in the cumulative concentrations achieved by peptones in this study, the ammonia inhibition does not seem to be a key factor for this cell line as seen with many others [39].

As shown in Table 1, the total amino acid contents of the peptones studied did not exceed about 80% in mass, with soy-derived peptones containing the lowest percentage of amino acids (about 50%). We can therefore suppose that the observed effects may be due at least in part to other nutrients than amino acids, which are possibly present in the hydrolysates. Previous studies also revealed that peptone hydrolysates may contain a variable proportion of other nutrients, such as sugars, lipids, vitamins, nucleic acids, and minerals [42-45].

All peptones tested in the present study had a similar molecular weight distribution, with over 80% of all fractions below 1 kDa (Table 1) and almost 100% below 10 kDa. Lower molecular weight fractions of plant peptones have been proven to better support cell growth and prolong cell viability, besides facilitating downstream product recovery [46]. 

The cultivation of rCHO cells in peptone-supplemented, serum-free media is a widely established cultivation method for the production of therapeutic proteins. The data presented in this study suggest that based on amino acid profile of peptones and its compatibility with basal media, cell-line tailored feeding strategy can be developed to improve the productivity of rCHO cell lines in basal media.

## References

[1] Heidemann R, Zhang C, Qi H, Larrick RJ, Rozales C, Park S (2000). The use of peptones as medium additives for the production of a recombinant therapeutic protein in high density perfusion cultures of mammalian cells. Cytotechnology.

[2] Merten OW (1999). Safety issues of animal products used in serum-free media. Dev Biol Stand.

[3] Chun BH, Kim JH, Lee HJ, Chung N (2007). Usability of size-excluded fractions of soy protein hydrolysates for growth and viability of Chinese hamster ovary cells in protein-free suspension culture. Bioresour Technol.

[4] Sung YH, Lim SW, Chung JY, Lee GM (2004). Yeast hydrolysate as a low-cost additive to serum-free medium for the production of human thrombopoietin in suspension cultures of Chinese hamster ovary cells. Appl Microbiol Biotechnol.

[5] Franek F, Hohenwarter O, Katinger H (2000). Preparation of defined peptide fractions promoting growth and production in animal cells cultures. Biotechnol Prog.

[6] Pham PL, Perret S, Doan HC, Cass B, St-Laurent G, Kamen A (2003). Large-scale transient transfection of serum-free suspension-growing HEK293 EBNA1 cells: peptone additives improve cell growth and transfection efficiency. Biotechnol Bioeng.

[7] Mendonca RZ, de Oliveira EC, Pereira CA, Lebrun I (2007). Effect of bioactive peptides isolated from yeastolate, lactalbumin and NZCase in the insect cell growth. Bioprocess Biosyst Eng.

[8] Pham PL, Perret S, Cass B, Carpentier E, St-Laurent G, Bisson L Transient gene expression in HEK293 cells: peptone addition posttransfection improves recombinant protein synthesis. Biotechnol Bioeng.

[9] Franek F, Eckschlager T, Katinger H (2003). Enhancement of monoclonal antibody production by lysine-containing peptides. Biotechnol Prog.

[10] Schlaeger EJ (1996). The protein hydrolysate, Primatone RL, is a cost-effective multiple growth promoter of mammalian cell culture in serum-containing and serum-free media and displays anti-apoptosis properties. J Immunol Methods.

[11] Shirsat N, Avesh M, English NJ, Glennon B, Al-Rubeai M (2013). Application of statistical techniques for elucidating flow cytometric data of batch and fed-batch cultures. Biotechnol Appl Biochem.

[12] Kim do Y, Chaudhry MA, Kennard ML, Jardon MA, Braasch K, Dionne B (2013). Fed-batch CHO cell t-PA production and feed glutamine replacement to reduce ammonia production. Biotechnol Prog..

[13] Mosser M, Chevalot I, Olmos E, Blanchard F, Kapel R, Oriol E (2013). Combination of yeast hydrolysates to improve CHO cell growth and IgG production. Cytotechnology.

[14] Kim JY, Kim YG, Han YK, Choi HS, Kim YH, Lee GM (2011). Proteomic understanding of intracellular responses of recombinant Chinese hamster ovary cells cultivated in serum-free medium supplemented with hydrolysates. Appl Microbiol Biotechnol.

[15] Zhang H, Wang HF, Liu MF, Zhang TF, Zhang JF, Wang XF (2013). Rational development of a serum-free medium and fed-batch process for a GS-CHO cell line expressing recombinant antibody. Cytotechnology.

[16] Mahboudi F, Abolhassan MR, Azarpanah A, Aghajani-Lazarjani H, Sadeghi-Haskoo MA, Maleknia S (2013). The role of different supplements in expression level of monoclonal antibody against human CD20. Avicenna J Med Biotechnol.

[17] Lai T, Yang Y, Kong S (2013). Advances in mammalian cell line development technologies for recombinant protein production. Pharmaceuticals (Basel).

[18] Beelen M, Zorenc A, Pennings B, Senden JM, Kuipers H, van Loon LJ (2011). Impact of protein coingestion on muscle protein synthesis during continuous endurance type exercise. Am J Physiol Endocrinol Metab.

[19] Thombre S, Gadgil M (2011). Increase in efficiency of media utilization for recombinant protein production in Chinese hamster ovary culture through dilution. Biotechnol Appl Biochem.

[20] Hegde S, Pant T, Pradhan K, Badiger M, Gadgil M (2012). Controlled release of nutrients to mammalian cells cultured in shake flasks. Biotechnol Prog.

[21] Davami F, Sardari S, Majidzadeh AK, Hemayatkar M, Barkhordari F, Enayati S (2011). A novel variant of t-PA resistant to plasminogen activator inhibitor-1; expression in CHO cells based on in silico experiments. BMB Rep.

[22] Davami F, Sardari S, Majidzadeh AK, Hemayatkar M, Barkhrdari F, Omidi M (2010). Expression of a novel chimeric truncated t-PA in CHO cells based on in silico experiments. J Biomed Biotechnol.

[23] Davami F, Barkhordari F, Alebouyeh M, Adeli A, Mahboudi F (2011). Combined TGE-SGE expression of novel PAI-1-resistant t-PA in CHO DG44 cells using orbitally shaking disposable bioreactors. J Microbiol Biotechnol.

[24] Muller N, Girard P, Hacker DL, Jordan M, Wurm FM (2005). Orbital shaker technology for the cultivation of mammalian cells in suspension. Biotechnol Bioeng.

[25] Stettler M, Jaccard N, Hacker D, De JM, Wurm FM, Jordan M (2006). New disposable tubes for rapid and precise biomass assessment for suspension cultures of mammalian cells. Biotechnol Bioeng.

[26] Bergmeyer HU (1974). Methods of Enzymatic Analysis.

[27] Medbo JI, Mamen A, Holt Olsen O, Evertsen F (2000). Examination of four different instruments for measuring blood lactate concentration. Scand J Clin Lab Invest.

[28] Butler M (2005). Animal cell cultures: recent achievements and perspectives in the production of biopharmaceuticals. Appl Microbiol Biotechnol.

[29] Kim SH, Lee GM (2009). Development of serum-free medium supplemented with hydrolysates for the production of therapeutic antibodies in CHO cell cultures using design of experiments. Appl Microbiol Biotechnol.

[30] Davami F, Baldi L, Rajendra Y, Wurm M (2014). Peptone Supplementation of Culture Medium Has Variable Effects on the Productivity of CHO Cells. Int J Mol Cell Med.

[31] Baycin-Hizal D, Tabb DL, Chaerkady R, Chen L, Lewis NE, Nagarajan H (2012). Proteomic analysis of Chinese hamster ovary cells. J Proteome Res.

[32] Burteau CC, Verhoeye FR, Mols JF, Ballez JS, Agathos SN, Schneider YJ (2003). Fortification of a protein-free cell culture medium with plant peptones improves cultivation and productivity of an interferon-gamma-producing CHO cell line. In vitro Cell Dev Biol Anim.

[33] Franek F (2004). Gluten of spelt wheat (Triticum aestivum subspecies spelta) as a source of peptides promoting viability and product yield of mouse hybridoma cell cultures. J Agric Food Chem.

[34] Jan DC, Jones SJ, Emery AN, al-Rubeai M (1994). Peptone, a low-cost growth-promoting nutrient for intensive animal cell culture. Cytotechnology.

[35] Keen MJ, Rapson NT (1995). Development of a serum-free culture medium for the large scale production of recombinant protein from a Chinese hamster ovary cell line. Cytotechnology.

[36] Ozturk SS, Riley MR, Palsson BO (1992). Effects of ammonia and lactate on hybridoma growth, metabolism, and antibody production. Biotechnol Bioeng.

[37] Kurano N, Leist C, Messi F, Kurano S, Fiechter A (1990). Growth behavior of Chinese hamster ovary cells in a compact loop bioreactor. 2. Effects of medium components and waste products. J Biotechnol.

[38] Hansen HA, Emborg C (1994). Influence of ammonium on growth, metabolism, and productivity of a continuous suspension Chinese hamster ovary cell culture. Biotechnol Prog.

[39] Dyring C, Hansen HA, Emborg (1994). Emborg . Observations on the influence of glutamine, asparagine and peptone on growth and t-PA production of Chinese hamster ovary (CHO) cells. Cytotechnology.

[40] Lee FW, Elias CB, Todd, P, Kompala DS (1998). Engineering chinese hamster ovary (CHO) cells to achieve an inverse growth–associated production of a foreign protein, β-galactosidase. Cytotechnology.

[41] Hansen HA, Emborg C (1994). Extra- and intracellular amino acid concentrations in continuous Chinese hamster ovary cell culture. Appl Microbiol Biotechnol.

[42] Michiels JF, Barbau J, De Boel S, Dessy S, Agathos SN, Schneider YJ (2011). Characterisation of beneficial and detrimental effects of a soy peptone, as an additive for CHO cell cultivation. Process Biochemistry.

[43] Michiels JF, Sart S, Schneider YJ, Agathos SN (2011). Effects of a soy peptone on γ-IFN production steps in CHO-320 cells. Process Biochemistry.

[44] Ballez JS, Mols J, Burteau C, Agathos SN, Schneider YJ (2004). Plant protein hydrolysates support CHO-320 cells proliferation and recombinant IFN-γ production in suspension and inside microcarriers in protein-free media. Cytotechnology.

[45] Chabanon G, Alves da CL, Farges B, Harscoat C, Chenu S, Goergen JL (2008). Influence of the rapeseed protein hydrolysis process on CHO cell growth. Bioresour Technol.

[46] Lee JY, Chun BH, Lee JH, Ahn J (2009). Chung N: Influence of mixed protein hydrolysates on the growth and viability of Chinese hamster ovary cells. J Korean Soc Appl Biol Chem.

